# Ultrasensitive Immunosensor for Prostate-Specific Antigen Based on Enhanced Electrochemiluminescence by Vertically Ordered Mesoporous Silica-Nanochannel Film

**DOI:** 10.3389/fchem.2022.851178

**Published:** 2022-03-03

**Authors:** Kai Ma, Yanyan Zheng, Lizhe An, Jiyang Liu

**Affiliations:** ^1^ Urology and Lithotripsy Center, Peking University People’s Hospital, Beijing, China; ^2^ Peking University Applied Lithotripsy Institute, Peking University, Beijing, China; ^3^ Key Laboratory of Surface and Interface Science of Polymer Materials of Zhejiang Province, Department of Chemistry, Zhejiang Sci-Tech University, Hangzhou, China

**Keywords:** immunosensor, electrochemiluminescence, ultrasensitive detection, prostate-specific antigen, vertically ordered mesoporous silica-nanochannel film

## Abstract

Ultrasensitive and specific detection of prostate-specific antigen (PSA) in complex biological samples is crucial for early diagnosis and treatment of prostate-related diseases. Immunoassay with a simple sensing interface and ultrahigh sensitivity is highly desirable. Herein, a novel electroluminescence (ECL) immunosensing platform is demonstrated based on the equipment of vertically ordered mesoporous silica-nanochannel films (VMSFs) with PSA antibody, which is able to realize ultrasensitive detection of PSA in human serum. Through the electrochemically assisted self-assembly (EASA) method, the VMSF is easily grown on an indium tin oxide (ITO) electrode in a few seconds. Owing to a large surface area and the negatively charged surface, VMSF nanochannels display strong electrostatic attraction to the positively charged ECL luminophores (tris(2,2-bipyridyl) dichlororuthenium (II), (Ru(bpy)_3_^2+^), leading to two orders-of-magnitude enhancement of ECL emission compared with that of the bare ITO electrode. The outer surface of the VMSF is functionalized with reactive epoxy groups, which further allows covalent attachment of PSA antibody (Ab) on the entry of nanochannels. As the combination of PSA with Ab decreases the ECL signal by hindering the mass transfer of ECL luminophores and coreactant, the developed immunosensor can achieve ultrasensitive detection of PSA ranging from 1 pg ml^−1^ to 100 ng ml^−1^ with a limit of detection (LOD) of 0.1 pg ml^−1^. Considering the antifouling ability of the VMSF, sensitive detection of PSA in human serum is also realized. The proposed nanochannel-based immunosensor may open up a new way for the facile development of the universal immunosensing platform for rapid and ultrasensitive detection of disease markers.

## 1 Introduction

Detection of disease-related biomarkers in complex biological samples is of great significance for the early diagnosis and treatment of diseases. (Li et al., 2015; Wang and Kan, 2021; Xu et al., 2021) For instance, prostate-specific antigen (PSA) is considered to be the most effective biomarker for clinical diagnosis of prostate-related diseases (e.g. rostatitis, enlarged prostate, nephritis, prostate polyps, and prostate cancer) because it can specifically reflect the condition of the prostate. (Liu et al., 2017; Yang et al., 2017; Yang et al., 2020) PSA is a single-chain polypeptide comprising 237 amino acid residues belonging to the human kallikrein family. As known, the concentration of PSA in the blood of a normal man is usually low (less than 4 ng/ml), but the PSA concentration in patients with prostate-related diseases is abnormally elevated. (Ma et al., 2016; Cao et al., 2018; Khoshfetrat et al., 2021) In the past few decades, various traditional techniques have been used for PSA detection, including enzyme-linked immunosorbent assay (ELISA), colorimetry, fluorescence, electrochemistry, chemiluminescence, and surface-enhanced Raman scattering (SERS). (Deng et al., 2015; Zhu et al., 2018; Hou et al., 2020) However, ultrasensitive detection of PSA in blood serum through a specific immunoassay platform with a simple sensing interface and high sensitivity is still a challenge.

Electroluminescence (ECL), which is generated with the electrochemical redox reaction of luminophores on the electrode surface, is an important and powerful analytical technology in bioassay owing to its fast detection, low background, high potential and spatial controllability, and wide dynamic response range. (Liu et al., 2015; Li et al., 2017; Zhang et al., 2019; Ma et al., 2020) In addition, the electrochemical excitation and simplified optical devices make ECL possible to be miniaturized in portable point-of-care testing (POCT). For instance, ECL of tris(2,2-bipyridyl) dichlororuthenium (II) (Ru(bpy)_3_^2+^) with tripropylamine (TPrA) as a coreactant has been widely used in most ECL systems to detect various samples. (Liu et al., 2015; Gu et al., 2020) In recent years, the rapid development of nanotechnology has greatly benefited ECL technology by further improving its sensitivity and universality. On the one hand, some nanomaterials (e.g. graphene quantum dots, gold nanoclusters, etc.) have been directly used as ECL emitters due to their high stability, good biocompatibility, and excellent luminescent characteristics. (Li et al., 2017; Zhang et al., 2019; Ma et al., 2020) On the other hand, nanomaterials with high surface area and porous structure promise great potential in the fabrication of an ultrasensitive ECL detection platform by loading a large number of luminophores.

Solid nanofilms (SNFs) have recently attracted extensive attention and displayed great potential in the fields of nanofluids, molecular sieves, nanoreactors, and biosensing because of their advantages of low cost, high stability, adjustable nanopores, and intelligent control of molecular transmission. (Huang et al., 2016; Cui et al., 2020; Cui et al., 2021; Duan et al., 2021; Garbayo et al., 2021) As an important class of SNFs, the vertically ordered mesoporous silica-nanochannel film (VMSF) is of particular interest because of its ordered and vertically arranged nanochannels, uniform and adjustable pore size distribution (usually 2–3 nm), controllable nanoscale thickness (ranging from 50 to 150 nm), excellent permeability property, and easy modification. (Nasir et al., 2016; Chen et al., 2019; Wang et al., 2020; Zhu et al., 2022) Owing to the capability of sieving molecules on the nanoscale, the VMSF-modified electrodes possess excellent antifouling ability in complex real samples and allow direct detection of analytes without complicated sample pre-treatment processes (e.g. separation). (Yan et al., 2016; Ding et al., 2020a; Ding et al., 2020b) It is also noteworthy that the VMSF offers great advantages for the fabrication of a universal ECL biosensor with ultrahigh sensitivity. On the one hand, open silica-nanochannels with high packing density (*>*10^12^ pores/cm^2^) endow the VMSF with high surface area and a large number of silanol groups (p*K*_a_ of ∼2). The abundant negatively charged sites on the surface of VMSF nanochannels favor accelerated mass transfer and enrichment of the positively charged luminophore (e.g., Ru(bpy)_3_^2+^) through strong electrostatic attraction, leading to significantly improved ECL sensitivity. (Zhou et al., 2015; Guo et al., 2016; Liu et al., 2016) On the other hand, silanol groups at the outer surface of the VMSF (also the entry of nanochannels) offer easy modification with silane-coupling reagents containing reactive groups (e.g., epoxy or amino group), allowing further immobilization of biomacromolecules with recognitive properties. Thus, it is highly encouraged to adopt VMSF-modified electrodes to fabricate the ECL immunosensor with ultrahigh sensitivity.

**FIGURE 1:**
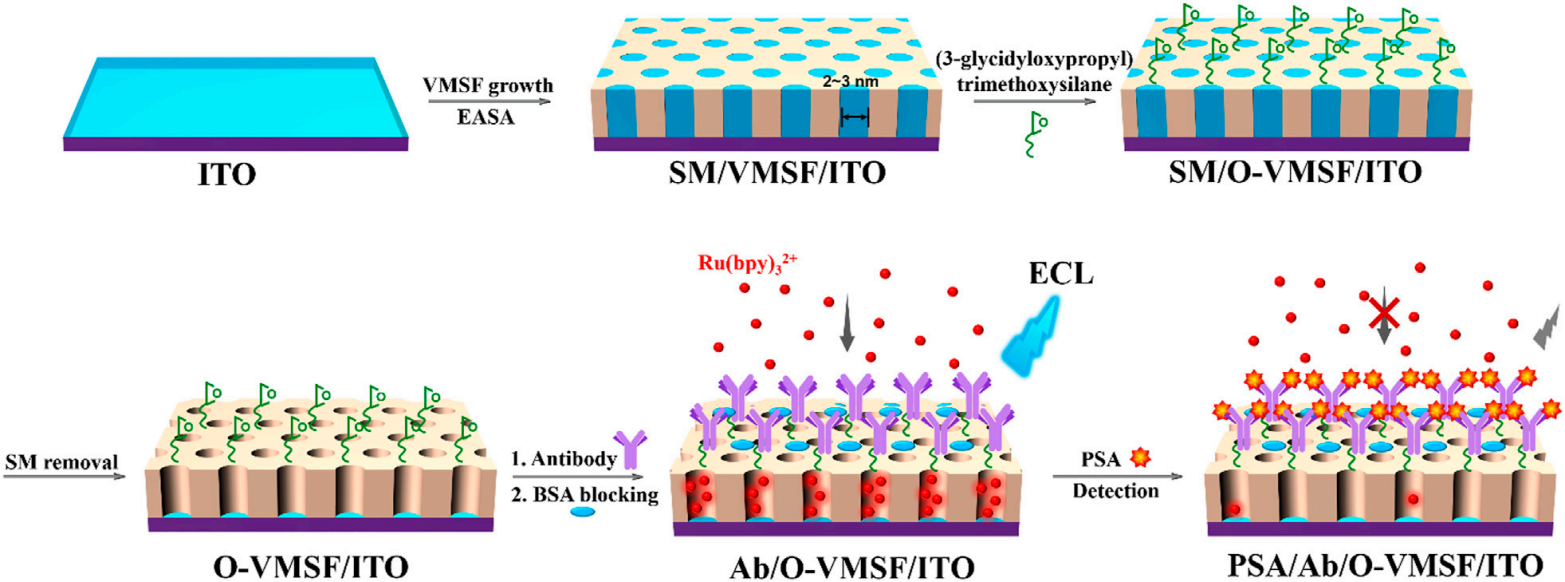
Schematic illustration for the fabrication of the immunosensing interface on the VMSF-modified electrode and the following label-free ECL detection of PSA.

In this study, we present a novel immunosensor for ultrasensitive detection of PSA based on enhanced ECL by the VMSF nanochannel. As illustrated in Figure 1, the VMSF is fast and easily grown on an indium tin oxide (ITO) electrode through an electrochemically assisted self-assembly (EASA) method. Owing to the significant enrichment of the positively charged luminophores (Ru(bpy)_3_^2+^) by VMSF nanochannels, the VMSF/ITO electrode displays a significantly enhanced ECL signal, which is 100 times higher than that of the bare ITO electrode. To fabricate a specifically recognitive interface, the outer surface of the VMSF is modified with reactive epoxy groups to further allow covalent attachment of PSA antibody (Ab). The feasibility of the developed immunosensor is validated by ECL detection of PSA based on the decrease in the mass transfer of Ru(bpy)_3_^2+^ luminophore after the specific binding of PSA and Ab. Combined with the excellent antifouling performance of the VMSF, the immunosensor can also be used for the ultrasensitive and selective detection of PSA in serum.

## 2 Materials and Methods

### 2.1 Chemicals and Materials

Prostate-specific antigen (PSA) and mouse anti-human PSA monoclonal antibody (Ab) were purchased from Beijing KEY-BIO Biotech Co., Ltd. (China). Tris(2,2-bipyridyl) dichlororuthenium (II) hexahydrate (Ru(bpy)_3_Cl_2_·6H_2_O) and hexaammineruthenium (III) chloride (Ru(NH_3_)_6_Cl_3_) were obtained from Sigma-Aldrich (United States). Tetraethoxysilane (TEOS), cetyltrimethylammonium bromide (CTAB), potassium ferricyanide (K_3_[Fe(CN)_6_]), bovine serum albumin (BSA), (3-glycidyloxypropyl) trimethoxysilane, and NaOH were obtained from Aladdin (China). Phosphate-buffered saline (PBS, 0.01 M, pH 7.4) was prepared by mixing NaH_2_PO_4_ and Na_2_HPO_4_ in a certain ratio. Human blood serum (healthy man) for real sample analysis was provided by Hangzhou Institute of Occupational Diseases (Hangzhou, China). ITO glass (<17Ω/square, thickness: 100 ± 20 nm) was obtained from Zhuhai Kaivo Optoelectronic Technology (China). To obtain a clean and negatively charged surface, the ITO glass was cleaned by NaOH solution (1 M) and then ultrasonically treated in acetone, ethanol, and deionized water. All other chemicals were of analytical grade. Ultrapure water (18.2 MΩ cm) was used throughout the study.

### 2.2 Measurements and Instrumentations

Scanning electron microscopy (SEM) images were collected on a SU8100 microscope (Hitachi, Japan) at an acceleration voltage of 5 kV. Transmission electron microscope (TEM) investigation was performed on a JEM-2100 microscope (JEOL Ltd., Japan) using an acceleration voltage of 200 kV. Electrochemical investigations including electrochemical impedance spectroscopy (EIS) and cyclic voltammetry (CV) were carried out on an Autolab electrochemical workstation (PGSTAT302N, Metrohm, Switzerland). Electrochemiluminescence (ECL) measurements were performed in a quartz cell using an MPI A multifunctional ECL analyzer (Xi’an Remax Electronic Science and Technology Co. Ltd.). The voltage of the photomultiplier tube was set at 400 V. A conventional three-electrode system was used for both electrochemical and ECL measurements. Briefly, a bare or modified ITO electrode was applied as the working electrode. A platinum disk (1 cm × 1 cm) was used as the counter electrode, and an Ag/AgCl electrode (saturated with KCl) was used as the reference electrode.

### 2.3 Preparation of Vertically Ordered Mesoporous Silica-Nanochannel Film–Modified Indium Tin Oxide Electrode

As previously reported, (Zhu et al., 2022) the VMSF-modified ITO electrode was prepared by growing the VMSF on the conducting ITO electrode by an electrochemically assisted self-assembly (EASA) method. Briefly, the precursor solution was prepared by adding TEOS (13.6 mM) and CTAB (4.35 mM) in the mixture (v:v = 1:1) of ethanol and NaNO_3_ solution (0.1 M). After adjusting the pH value to 3.0 with hydrochloric acid (HCl, 6 M), the solution was aged for 2.5 h under stirring. A cleaned ITO was then immersed into the precursor solution, and a constant current (−350 μA) was applied for 10 s. After that, the electrode was quickly taken out and thoroughly washed with ultrapure water. After drying with nitrogen (N_2_), the obtained electrode was aged at 120°C overnight. As the CTAB surfactant micelle (SM) still existed in the VMSF channel, the obtained electrode was recorded as SM@VMSF/ITO. The SM could be removed by stirring in 0.1 M HCl/ethanol solution for 5 min.

### 2.4 Fabrication of the Immunosensor

The fabrication of the immuno-recognitive interface on the VMSF-modified ITO electrode included three steps. First, reactive epoxy groups were introduced on the outer surface of the VMSF. To avoid modification of the interior surface of VMSF nanochannels, the SM@VMSF/ITO with SM-blocked nanochannels was immersed in (3-glycidyloxypropyl) trimethoxysilane (2.26 mM in ethanol) for 1 h to introduce epoxy groups on the outer surface of the VMSF. The electrodes were then thoroughly washed with ultrapure water. Second, the SM in the VMSF was removed to obtain open nanochannels, and the obtained electrode is named O-VMSF/ITO. Third, PSA antibody (Ab) was covalently immobilized through incubation with Ab (10 μg/ml) at 37°C for 90 min followed by thorough rinsing with PBS (0.01 M, pH 7.4). Then, the electrode was incubated with BSA solution (1%, w/w) for 90 min to block the nonspecific binding sites to produce a PSA immunosensor, which was denoted as Ab/O-VMSF/ITO.

### 2.5 Electroluminescence Determination of Prostate-Specific Antigen

The Ab/O-VMSF/ITO sensor was incubated with different concentrations of PSA (antigen) at 37°C for 45 min. The ECL intensity of the immunosensor before and after PSA binding was measured. Briefly, Ab/O-VMSF/ITO or PSA/Ab/O-VMSF/ITO electrodes were immersed in Ru(bpy)_3_^2+^ solution (10 μM, in 0.01 M PBS, pH = 7.4) containing TPrA (3 mM) for 30 min to enrich the ECL luminophores. Then, ECL signals of the electrodes were recorded when continuous potential scanning between 0 to 1.25 V was applied at a scanning rate of 100 mV/s. For real sample analysis, human serum (healthy male) was diluted 50 times with PBS (0.01 M, pH 7.4) and directly determined using the developed immunosensor.

## 3 Results and Discussion

### 3.1 Facile Preparation of Vertically Ordered Mesoporous Silica-Nanochannel Film–Modified Electrode and the Significantly Enhanced Electroluminescence Signal

Figure 1 illustrates the fabrication of the immunosensing interface on the VMSF modified electrode and the following label-free electrochemiluminescence (ECL) detection of prostate-specific antigen (PSA). The first step for the construction of an immunosensor is to rapidly grow vertically ordered mesoporous silica-nanochannel films (VMSFs) on an ITO by the electrochemically assisted self-assembly (EASA) method. EASA allows quick deposition of the VMSF (within 10 s) under kinetic control at room temperature. When the cathode potential is applied to the ITO electrode, the protons and water molecules are reduced, causing a local increase in the pH at the electrode/solution interface. It is beneficial to the condensation of the siloxane precursor (tetraethoxysilane, TEOS) around the surfactant (cetyltrimethylammonium bromide, CTAB) micelle (SM) template. After the VMSF grows, the SM is found in the electrode (SM@VMSF/ITO), and it can be easily removed by soaking in hydrochloric acid–ethanol to obtain the VMSF-modified electrode (VMSF/ITO) with open nanochannels.

**FIGURE 2:**
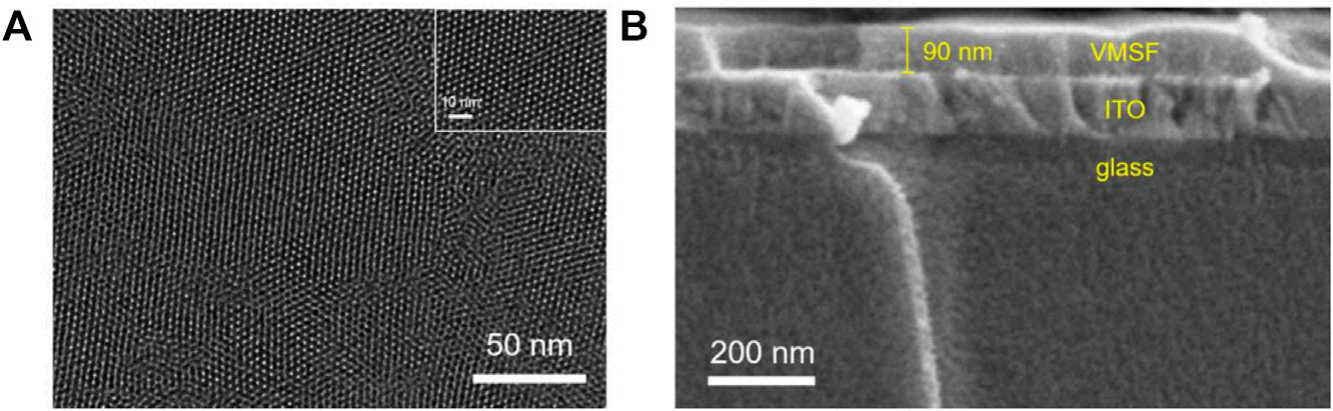
**(A)** Top-view TEM images of the VMSF separated from the ITO electrode at different magnification. **(B)** Cross-sectional SEM image of the VMSF/ITO.

Scanning electron microscopy (SEM) and transmission electron microscopy (TEM) are used to confirm that the VMSF could be successfully modified on the ITO electrode. As shown in Figure 2A, the VMSF separated from the ITO electrode has highly uniform and ordered nanopores with a diameter of about 2–3 nm. The pore density is ∼7.4 × 10^12^/cm^2^, which corresponds to porosity of ∼38%. The cross-sectional SEM image of the VMSF/ITO shows a three-layer structure, corresponding to VMSF, ITO, and glass (Figure 2B). In addition, the VMSF is a uniform film with a thickness of about 90 nm.

**FIGURE 3:**
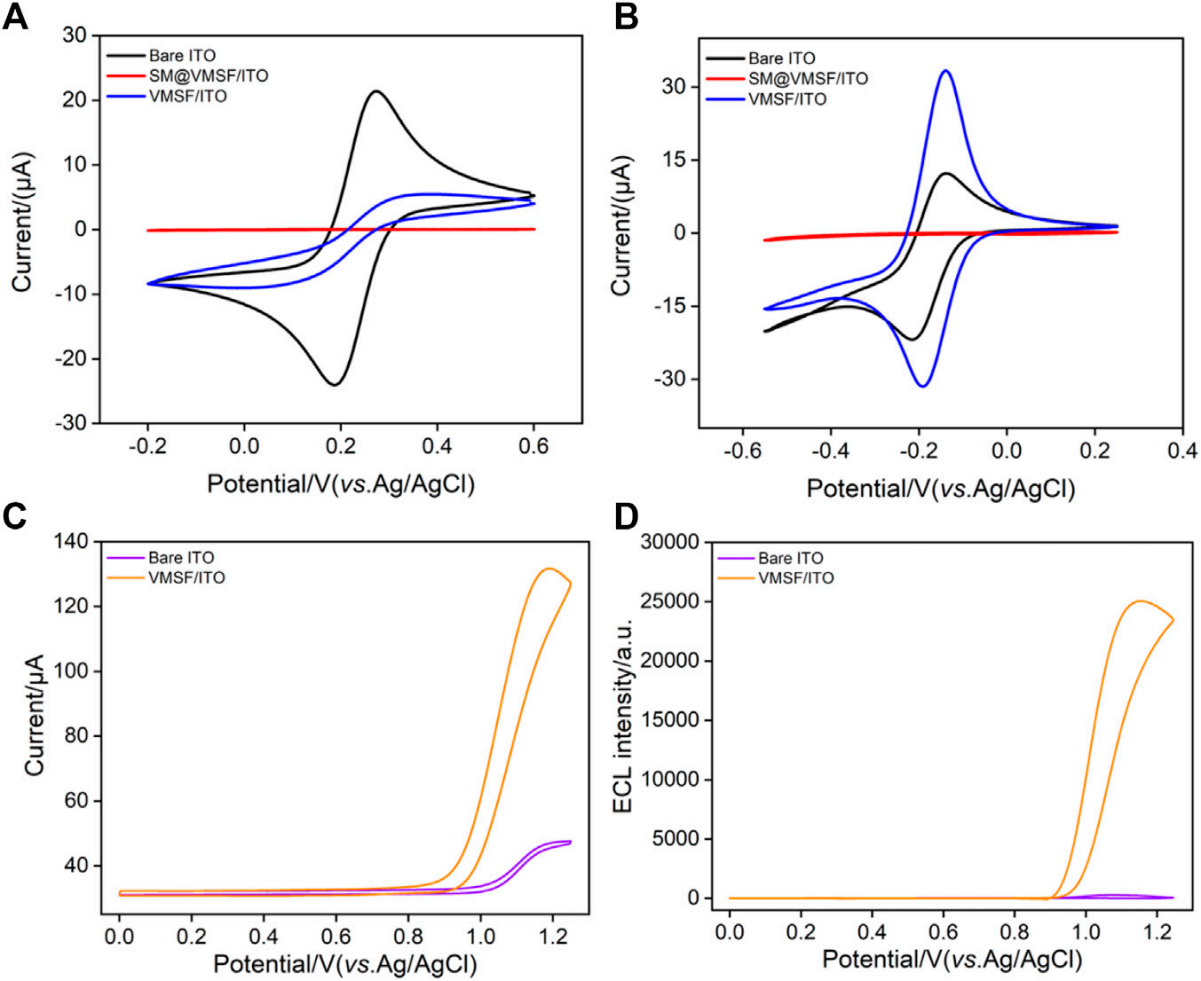
Cyclic voltammetric curves of **(A)** K_3_[Fe(CN)_6_] (0.5 mM) or **(B)** Ru(NH_3_)_6_Cl_3_ (0.5 mM) on the bare ITO, SM@VMSF/ITO, and VMSF/ITO electrodes in KHP solution (0.05 M, pH = 7.4). The scan rate is 50 mV s^−1^.Cyclic voltammetric curves **(C)** and ECL intensity-potential curves **(D)** obtained at the bare ITO or VMSF/ITO in 0.1 M PBS (pH 7) containing Ru(bpy)_3_^2+^ (10 μM) and TPrA (3 mM).

To prove the integrity and permeability of the VMSF, the electrochemical behaviors of the standard redox probes on different electrodes are investigated (Figures 3A,B). As shown, almost no electrochemical signals of both cationic (Ru(NH_3_)_6_^3+^) and anionic (Fe(CN)_6_^3−^) probes are observed on the electrode containing the micelle (SM@VMSF/ITO). Thus, the full coverage of SM blocks nanochannels and inhibits the electron transfer between the probe and the electrode substrate. This phenomenon proves that the grown VMSF is intact with almost no defects. When the micelles in the nanochannel are removed, the electrochemical signals of the two probes can be detected on the VMSF/ITO, indicating the entrance of the redox probe into the nanochannel. Compared with the bare ITO electrode, the VMSF/ITO displays a suppressed signal of anionic Fe(CN)_6_^3−^. On the contrary, a significantly higher current of cationic Ru(NH_3_)_6_^3+^ is observed, indicating a remarkable enrichment effect. This charge-selective permeability of the VMSF is attributed to the anionic nature of the silica walls generated by the abundant silanol groups (p*K*_a_ ∼ 2). Owing to the large surface-to-volume ratio of the VMSF and high load capacity, the VMSF offers accelerated mass transport and significant amplification characteristics toward cationic probes.

Among the developed ECL emitters, tris(2,2-bipyridyl) dichlororuthenium (II) (Ru(bpy)_3_^2+^) is the most extensively investigated and commercially used owing to its good chemical stability, high ECL quantum yield, and water solubility. The electrochemical and ECL signals of Ru(bpy)_3_^2+^ on the bare ITO or VMSF/ITO are compared with those of tripropylamine (TPrA) as the coreactant. As shown in Figure 3C, a significant anodic current wave is observed on the VMSF/ITO at potentials more positive than 0.9 V. In comparison with the bare ITO, the VMSF/ITO displays higher current and lower electrochemical oxidation potential, indicating the facilitated charge transfer of Ru(bpy)_3_^2+^ on the underlying electrode. The corresponding ECL signal can be observed in Figure 3D. Even at low voltage of the photomultiplier tube (400 V), the ECL signal on the VMSF/ITO electrode is two orders-of-magnitude (100 times) higher than that of the bare ITO electrode, indicating significant enhancement of ECL emission. Owing to the ultrasmall diameter (2–3 nm) and the negatively charged surface, the VMSF displays a strong electrostatic attraction to the positively charged Ru(bpy)_3_^2+^, which remarkably increases its concentration on the electrode surface, resulting in effective signal amplification.

### 3.2 Fabrication of the Immunosensing Interface on Vertically Ordered Mesoporous Silica-Nanochannel Films/Indium Tin Oxide Electrode

As a biosensing technology based on the specific recognition between antigen (Ag) and antibody (Ab), immunosensors have attracted wide attention due to their high specificity and sensitivity. With the recent development of bioengineering technology, antibodies toward various microorganisms, cell surface antigens, or protein antigens can be easily developed. This greatly expands the application of immunosensors in the fields of biological analysis, clinical diagnosis, etc. The VMSF itself is inert without biosensing capability. The facile method to fabricate the immunosensor by equipping the VMSF with antibody can greatly expand the application of nanochannel-based sensors. Although antibodies are too large to be introduced into the VMSF nanochannels, they could be easily immobilized on the outer surface of the VMSF, that is, entrances of the nanochannels. As illustrated in Figure 1, the antibody is immobilized at the nanochannel entry, giving rise to bio-recognitive function. The abundant hydroxyl groups at the VMSF surface favor the modification with the silane-coupling reagent with reactive groups. As shown, the VMSF is modified with (3-glycidyloxypropyl) trimethoxysilane to functionalize its surface with reactive epoxy groups, which further allows covalent attachment of the PSA antibody. In order to make the modification of epoxy groups only occur on the outer surface of the VMSF without changing the surface of nanochannels, SM@VMSF/ITO electrodes with nanochannels blocked with the SM are used for the modification. Then, the SM is removed to obtain an electrode with rich epoxy groups on the entry of nanochannels (O-VMSF/ITO). Compared with -COOH or -NH_2_ groups, that could be used to covalently link antibodies with catalysis (e.g. 1-(3-dimethylaminopropyl)-3-ethylcarbodiimide hydrochloride/N-hydroxysuccimide, EDC/NHS) or further modification (e.g. glutaraldehyde linker for -NH_2_ groups), epoxy groups offer a simple process for the covalent immobilization of antibodies without additional catalysts or modification.

**FIGURE 4:**
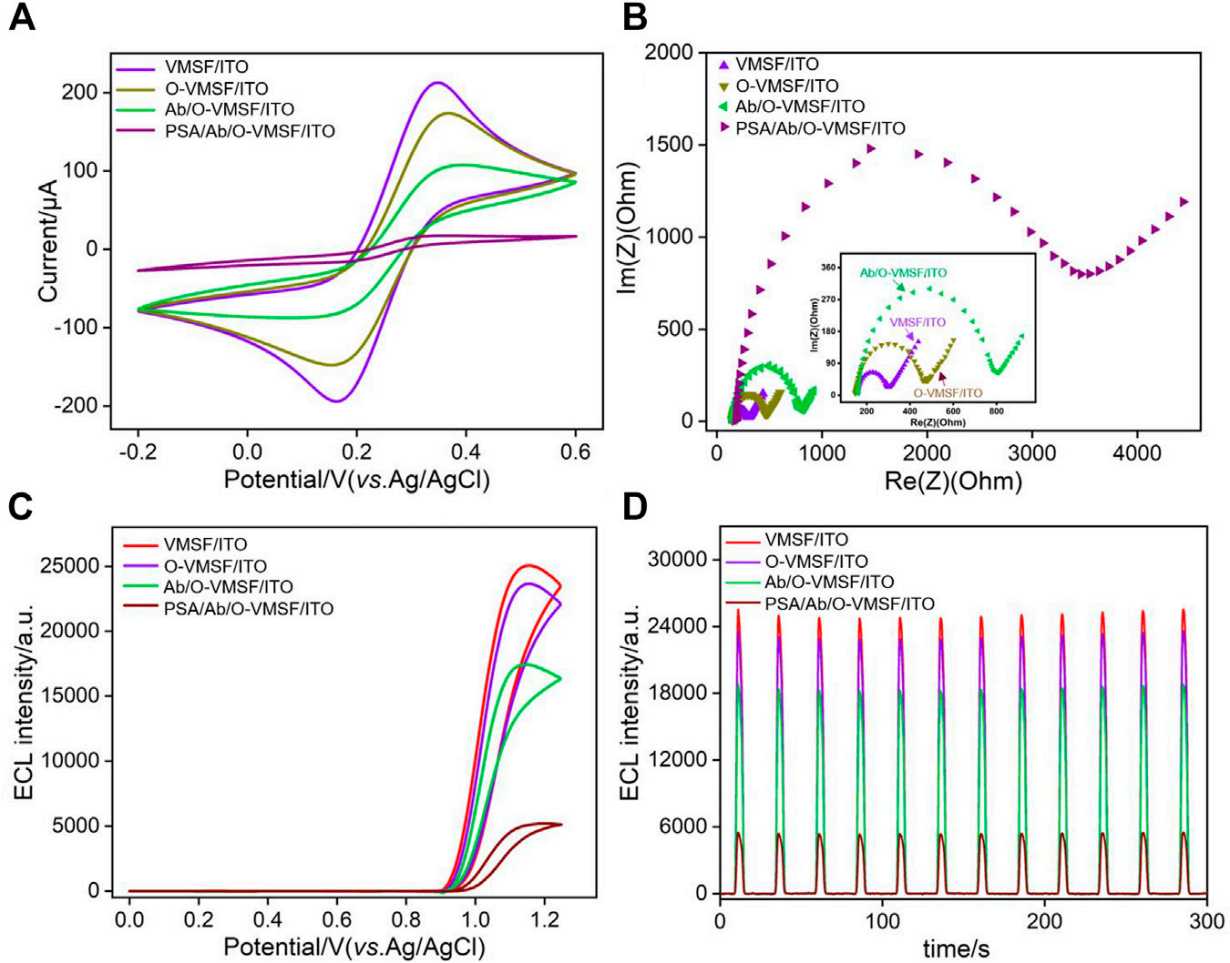
Cyclic voltammetric curves **(A)** and Nyquist plots **(B)** recorded on different electrodes in 5 mM K_3_[Fe(CN)_6_]/K_4_[Fe(CN)_6_] (1: 1) containing 0.1 M KCl. ECL intensity-potential curves **(C)** and ECL intensity-curves **(D)** obtained on different electrodes.

Cyclic voltammetry (CV) and electrochemical impedance spectroscopy (EIS) are used to investigate the changes occurring at the electrode surface during the fabrication of the immunosensor. As shown in Figure 4A, voltammograms recorded at the O-VMSF/ITO using [Fe(CN)_6_]^4−/3−^ as the redox probe is similar to that on the VMSF/ITO, indicating that the epoxy group modification does not significantly affect the properties of the nanochannels. When Ab is covalently immobilized and the nonspecific binding site is blocked by BSA, the redox current response of [Fe(CN)_6_]^4−/3−^ on the Ab/O-VMSF/ITO electrode is significantly reduced owing to the increased interface resistance generated by the nonconductive antibody. After incubation of the Ab/O-VMSF/ITO with PSA, the redox signal of the probe on the electrode is quite low, proving the formation of an antigen–antibody complex. Similar conclusions could be deduced from EIS measurements as illustrated in Figure 4B. As shown, the electron transfer resistance (*R*_et_), which is related to the semicircle diameter of each curve, is similar before and after modification with epoxy groups. Consecutive increase of the impedance value is obtained due to immobilization of Ab and the following BSA blocking because the protein layer acts as the inert electron and hinders the electron transfer. A significant increase in impedance value is observed when the Ab/O-VMSF/ITO electrode is incubated with PSA, indicating the successful capture of the antigen at the immuno-recognitive interface.

In order to verify the application of the as-prepared immunosensor in ECL detection, the ECL intensity-potential and intensity-time curves of Ru(bpy)_3_^2+^ are also investigated in presence of the TPrA coreactant. As shown in Figures 4C,D, strong ECL responses with similar intensity are obtained on both VMSF/ITO and O-VMSF/ITO electrodes, further proving that the group modification of the outer surface of the VMSF does not significantly affect the enrichment of positively charged luminophores by nanochannels. After Ab immobilization and BSA blocking, the ECL intensity of the Ab/O-VMSF/ITO electrode decreases. Owing to the ultrasmall pore size and high pore density of the nanochannels, the surface area of the nanochannels is significantly higher than that of the outer surface of the VMSF. Thus, the change of surface charge upon the modifications of biochemical species is not remarkable. The decreased ECL signal is due to the hindrance of the diffusion of the ECL luminophores and the coreactant to the electrode surface. Subsequently, the combination of PSA and Ab leads to a significant decrease in ECL intensity because the diffusion of Ru(bpy)_3_^2+^ and TPrA is further blocked. In addition, the ECL intensity of each electrode under continuous scanning exhibits a low relative standard deviation (RSD, less than 4%), indicating excellent stability.

**FIGURE 5:**
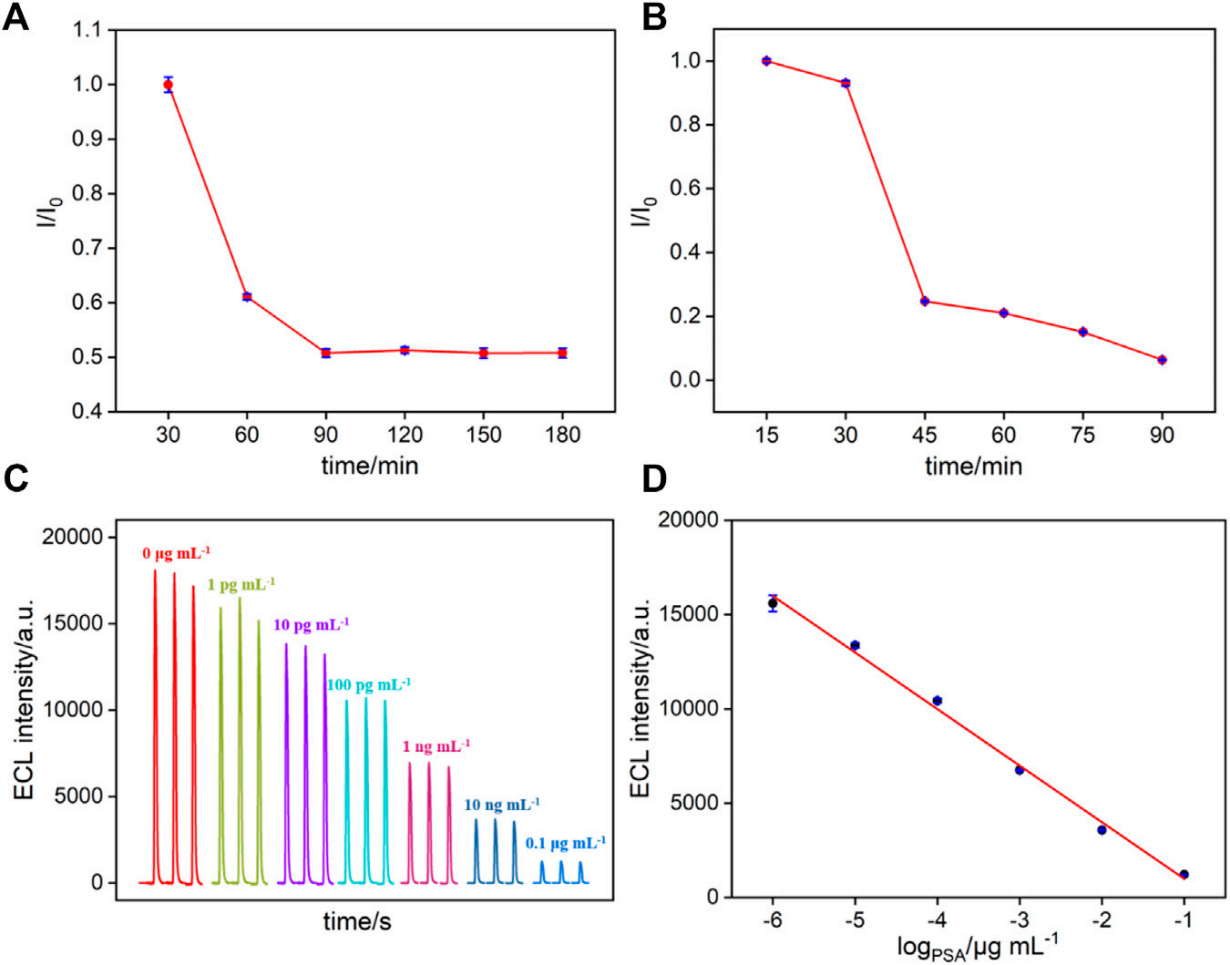
**(A–B)** The effect of antibody immobilization **(A)** or **(B)** PSA incubation time **(B)** on relative ECL signal ratio. **(C)** ECL response of the immunosensor in the presence of different concentrations of PSA. **(D)** Calibration curve for the detection of PSA.

The optimal conditions for preparation of an immunosensor, including the incubation time for antibody immobilization and reaction time for antigen–antibody interaction, are determined. The ECL intensity on the O-VMSF/ITO electrode before and after incubation with Ab for different time is investigated. As shown in Figure 5A, the relative ECL signal reaches a plateau when the incubation time for antibody immobilization is 90 min. In addition, when the interaction between the antigen and antibody is less than 45 min, the ECL signal decreases significantly with prolonged incubation time (Figure 5B). Thus, 90 min for antibody immobilization and 45 min for antigen–antibody interaction are chosen for further investigations.

### 3.3 Label-free Electroluminescence Determination of Prostate-Specific Antigen

The developed immunosensor is applied for label-free ECL determination of PSA based on the decrease in the mass transfer of the Ru(bpy)_3_^2+^ luminophore after specific binding of PSA and Ab (Figure 1). The analytical performance is evaluated by incubation with different concentrations of target PSA. As revealed in Figure 5C, the ECL intensity of the immunosensor gradually decreased with the increasing concentration of PSA. The calibration curve shows a good linear relationship between ECL intensity (*I*_ECL_) and the logarithmic value of PSA concentration (logC_PSA_) ranging from 1 pg ml^−1^ to 100 ng ml^−1^ (*I*_ECL_ = −2999 logC_PSA_+411.2, *R* ^2^ = 0.995, Figure 5D). The limit of detection (LOD) is calculated to be 0.1 pg ml^−1^ (*S*/*N* = 3). Supplementary Table S1 shows the comparison of ECL detection of PSA using different modified electrodes (in *Supporting Information*, SI). The LOD is lower than that obtained from the gold nanoparticle–modified glassy carbon electrode (AuNPs/GCE) (Wang and Kan, 2021), potassium niobate-Au nanoparticles@bismuth sulfide–modified GCE (KNbO_3_-Au NPs@Bi_2_S_3_/GCE) (Li et al., 2015), ferrocene–graphene sheets and Au-CdS flower-like 3D assembly–modified GCE (Fc-GNs/Au-CdS flower-like 3D assemblies/GCE) (Yang et al., 2017), Au nanoparticle/graphene quantum dots-poly(etherimide)-graphene oxide–modified GCE (AuNP/GQDs-PEI-GO/GCE) (Yang et al., 2020), EuPO_4_-modified GCE (Ma et al., 2016), gold nanorods functionalized graphene oxide multilabeled with glucose oxidase/electrodeposited gold–modified GCE (GO@AuNRs-GOD/DPAu) (Cao et al., 2018), AuNP-decorated MoS_2_/SiO_2_ nanoparticles labeled with glucose oxidase–modified GCE (MoS_2_-AuNPs/SiO_2_-GOD) (Hou et al., 2020), luminol loaded within the MIL-53(Fe)-NH_2_/nitrogen-doped graphene-coated Cu foam/bipolar electrode (L@MIL-53(Fe)-NH_2_/fCu/N-GN/RuNPs/BPE) (Khoshfetrat et al., 2019), and gold nanoprobe consisting of a peptide with a ruthenium(Ⅱ)- and nafion-modified GCE (AuNP peptide-Ru1/nafion/GCE) (Zhe et al., 2019), but higher than that obtained on amino-modified multiwalled carbon nanotubes/nafion- and Ru(bpy)_3_^2+^-modified GCE (MWCNTs-NH_2_@N/Ru(bpy)_3_^2+^/GCE). (Liu et al., 2017)

### 3.4 Selectivity, Reproducibility, and Stability of the Immunosensor

**FIGURE 6:**
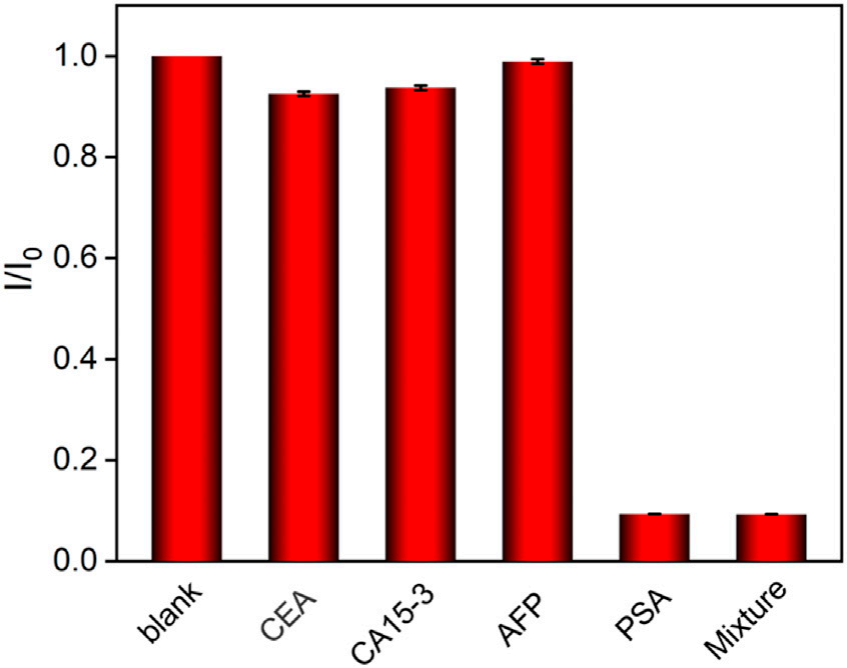
Relative ratio of ECL intensity before (I_0_) and after (I_0_) incubation with buffer (blank), CEA (500 ng ml^−1^), CA15-3 (500 ng ml^−1^), AFP (500 ng ml^−1^), PSA (0.1 ng ml^−1^), or protein mixture (0.1 ng ml^−1^ PSA + 500 ng ml^−1^ other proteins).

The specific recognition capacity of the developed immunosensor was studied by performing detection of PSA or other interfering tumor biomarkers, including carcinoembryonic antigen (CEA), carbohydrate antigen 15-3 (CA 15-3), and alphafetoprotein (AFP). Single tumor biomarker or their mixture is detected using the Ab/O-VMSF/ITO electrode. As shown in Figure 6, the obvious ECL decrease is only observed when the immunosensor is incubated with the PSA and the mixture. Other tumor biomarkers lead to no significant change in the ECL responses, indicating high selectivity for PSA detection. To evaluate the reproducibility of the immunosensor, five sensing electrodes are prepared in parallel, and their responses are investigated by measuring the ECL response toward 10 ng ml^−1^ PSA. All electrodes exhibit a similar analytical response, and a relative standard deviation of 3.2% is revealed, demonstrating high reproducibility. After 15 days of storage at 4°C, the analytical response of the immunosensor to 10 ng ml^−1^ PSA remained 97.2% of the initial signal, indicating high stability.

### 3.5 Real Sample Analysis

The feasibility of the developed immunosensor for practical application is evaluated by determining the concentration of the PSA in complex biological samples. A human serum sample of a healthy man is measured. The PSA concentration detected using the proposed immunosensor (1.48 ng ml^−1^) is in acceptable agreement with that obtained by the ROCHE ELISA ECL analyzer (1.51 ng ml^−1^). In addition, different concentrations of standard PSA solutions are artificially added into serum samples to simulate cancer patients with high PSA concentrations. Satisfactory recoveries of PSA ranging from 98.8% to 102.3% are obtained in this standard addition method, indicating good accuracy (Supplementary Table S2 in SI). The results demonstrate the practicability of the immunosensor for potential clinical analysis of PSA.

## 4 Conclusion

In summary, we have developed a novel label-free immunosensor based on vertically ordered mesoporous silica-nanochannel films (VMSFs), which is able to perform ultrasensitive detection of prostate-specific antigen (PSA) with high selectivity. Owing to the ultrasmall diameter and negatively charged surface, the VMSF nanochannels display strong electrostatic attraction to the positively charged ECL luminophores, leading to two orders-of-magnitude enhancement of ECL emission. The outer surface of the VMSF is functionalized with reactive epoxy groups, which further allows covalent attachment of the PSA antibody on the entry of nanochannels. The combination of PSA with Ab hinders the mass transfer of the ECL luminophores and coreactant, resulting in ultrasensitive detection of PSA ranging from 1 pg ml^−1^ to 100 ng ml^−1^ with an LOD of 0.1 pg ml^−1^. In combination with the antifouling ability of the VMSF, the developed immunosensor exhibits potential in PSA detection in complex biological samples. The VMSF-based immunosensor might open a new way for the facile fabrication of biosensing platforms with ultrahigh sensitivity and good antifouling performance.

## Data Availability

The original contributions presented in the study are included in the article/Supplementary Material; further inquiries can be directed to the corresponding authors.
