# Supplementary material for: Ultrasensitive Immunosensor for Prostate-Specific Antigen Based on Enhanced Electrochemiluminescence by Vertically Ordered Mesoporous Silica-Nanochannel Film
Source: Front Chem. 2022 Mar 3;10:851178. doi: 10.3389/fchem.2022.851178 (PMC8927089; doi:10.3389/fchem.2022.851178)
Supplement: Supplementary file 1 [file DataSheet1.doc]

Table S1 Comparison between ECL detection of PSA using different modified electrode.

| **Electrode** | **Detection range**  **ng/mL** | **LOD pg/mL** | **Ref.** |
| --- | --- | --- | --- |
| AuNPs/GCE | 5×10^-3^-50 | 3.0 | 2 |
| KNbO_3_-Au NPs@Bi_2_S_3_/GCE | 0.005-5 | 3.0 | 3 |
| MWCNTs-NH_2_@N/Ru(bpy)_3_^2+^/GCE | 10^-4^-20 | 3.5×10^-2^ | 4 |
| Fc-GNs/Au-CdS flower-like 3D assemblies/GCE | 10^-3^-25 | 0.38 | 5 |
| AuNP/GQDs-PEI-GO/GCE | 10^-3^-100 | 0.44 | 6 |
| EuPO_4_/GCE | 5×10^-4^-80 | 0.17733 | 8 |
| GO@AuNRs-GOD/DPAu/GCE | 5×10^-4^-5 | 0.17 | 9 |
| MoS_2_-AuNPs/SiO_2_-GOD/GCE | 5×10^-4^-10 | 0.2 | 12 |
| L@MIL-53(Fe)-NH_2_/fCu/N-GN/RuNPs/BPE | 10^-3^-300  5×10^-3^-200 | 0.2  0.1 | 33 |
| AuNP-peptide-Ru1/nafion/GCE | 10^-3^-1 | 0.4 | 34 |
| O-VMSF/ITO | 10^-3^-100 | 0.1 | Our work |

AuNPs/GCE, gold nanoparticle modified glassy carbon electrode; KNbO_3_-Au NPs@Bi_2_S_3_/GCE, potassium niobate-Au nanoparticles@bismuth sulfide modified glassy carbon electrode; MWCNTs-NH_2_@N, amino-modified multiwall carbon nanotubes/Nafion; Fc-GNs, ferrocene-graphene sheets; AuNP/GQDs-PEI-GO, Au nanoparticle/graphene quantum dots-poly(etherimide)-graphene oxide; GO@AuNRs-GOD/DPAu, gold nanorods functionalized graphene oxide multilabeled with glucose oxidase/electrodeposited gold, MoS_2_-AuNPs/SiO_2_-GOD, AuNPs decorated MoS_2_/SiO_2_ nanoparticles labeled with glucose oxidase; L@MIL-53(Fe)-NH_2_/fCu/N-GN/RuNPs/BPE, luminol loaded within the MIL-53(Fe)-NH_2_/nitrogen-doped graphene-coated Cu foam/bipolar electrode; AuNP-peptide-Ru1/nafion/GCE, gold nanoprobe consisted of a peptide with a ruthenium(Ⅱ).

Table S2 PSA detection in human serum.

| **Sample** | **Added PSA**  **(ng/mL)** | **Added PSA**  **(ng/mL)** | **RSD**  **(%, n=3)** | **Recovery**  **(%)** |
| --- | --- | --- | --- | --- |
| Human Serum | 5.00 | 4.97 | 1.6 | 99.4 |
|  | 15.00 | 15.35 | 0.8 | 102.3 |
|  | 30.00 | 29.64 | 1.3 | 98.8 |
